# The Importance of Toll-like Receptor 9 Expression on Monocytes and Dendritic Cells in the Context of Epstein–Barr Virus Infection in the Immunopathogenesis of Primary Glomerulonephritis

**DOI:** 10.3390/ijms231911796

**Published:** 2022-10-05

**Authors:** Iwona Smarz-Widelska, Sebastian Mertowski, Paulina Mertowska, Izabela Korona-Głowniak, Anna Hymos, Ewelina Grywalska, Wojciech Załuska

**Affiliations:** 1 Department of Nephrology, Cardinal Stefan Wyszyński Provincial Hospital in Lublin, 20-718 Lublin, Poland; 2 Department of Experimental Immunology, Medical University of Lublin, 4a Chodźki Street, 20-093 Lublin, Poland; 3 Department of Pharmaceutical Microbiology, Medical University of Lublin, 1 Chodźki Street, 20-093 Lublin, Poland; 4 Department of Nephrology, Medical University of Lublin, 8 Jaczewskiego Street, 20-954 Lublin, Poland

**Keywords:** Toll-like receptor 9, glomerulonephritis, IgA nephropathy, membranoproliferative glomerulonephritis, monocytes, dendritic cells, Epstein–Barr virus

## Abstract

Toll-like receptor 9 (TLR9) is activated by unmethylated cytosine-phosphate-guanosine (CpG) dinucleotides found in the genomes of pathogens such as Epstein–Barr virus (EBV). The aim of this study was to determine the role of TLR9 in the immunopathogenesis of IgA nephropathy (IgAN) and membranoproliferative glomerulonephritis (MPGN) in the context of Epstein–Barr virus (EBV) infection. For this purpose, the frequency of TLR9-positive monocytes and dendritic cells (DCs, i.e., BDCA-1; myeloid dendritic cells, and BDCA-2; plasmocytoid dendritic cells) was studied, and a quantitative analysis of the concentration of TLR9 in the serum of patients diagnosed with IgAN and MPGN was undertaken. Higher frequencies of TLR9-positive DCs and monocytes in IgAN and MPGN patients were observed as compared with the control group. Patients diagnosed with GN exhibited a higher percentage of BDCA-1+CD19− and BDCA-2+CD123+ DCs than patients in the control group. Moreover, serum TLR9 concentration was shown to be significantly correlated with EBV DNA copy number/µg DNA, IgG, IgM, serum albumin, total protein in 24-h urine collection test and the frequency of BDCA-2+CD123+ DCs in peripheral blood. Our findings confirm that TLR9 may be involved in the development of IgAN and MPGN.

## 1. Introduction

The pathogenesis of different types of primary glomerulonephritis (GN) is still not fully recognized. The literature results suggest that the disorders of the immune system, both in humoral and cellular response pathways, are directly involved in the development and progression of GN [1,2]. Although biopsy seems to be the gold standard in the diagnosis of nephropathy, the characterization of non-invasive and specific marker molecules to aid the diagnosis of nephropathy is still lacking. One of the factors increasing the diagnostic potential may be the option of determination of the role of Toll-like receptors (TLRs) in the immunopathogenesis of GN [3]. TLRs are key components of innate and adaptive immunity in the human body, responsible for controlling the host’s immune response to pathogens by recognizing microorganism-specific molecular patterns (PAMP) or damage-related molecular patterns (DAMP) [4,5]. One such example is Toll-like receptor 9 (TLR9), which is an intracellular receptor activated by unmethylated cytosine-phosphate-guanosine (CpG) dinucleotides (found in the genomes of pathogens such as Epstein–Barr virus (EBV) and endogenous mitochondrial DNA products released from the damaged cells [6,7]. Regarding the functional role of the kidneys, the presence of dendritic cells (DCs) and monocytes appears to be associated with the induction and regulation of inflammatory responses to the filtered antigen material; it also protects the kidneys against infections. According to the researchers, tissue-resident or -infiltrating DCs may constitute an important factor in the development and progression of kidney disease [8,9,10]. Currently, the information on four main types of these cells can be found in the literature: classical or myeloid (mDC, BDCA-1+CD19−) type, plasmacytoid (pDC, BDCA-2+CD123+) type, Langerhans cells (LC) and DCs derived from the monocytes (Mo-DCs). Both cDC1s and pDCsplay an extremely important role in the antitumor and antiviral response [11]. According to the scientific data, EBV may be one of the factors involved in the pathogenesis of kidney disease. Reports include lupus nephropathy and acute nephritis [12,13,14,15], and, accordingly with the results of our own observations also in course of GN [16]. 

The aim of this study was to determine the role of TLR9 in immunopathogenesis of IgA nephropathy (IgAN) and membranoproliferative glomerulonephritis (MPGN) as potentially diagnostically useful molecules and to review their role in EBV incidence in patients with GN. For this purpose, the presence of TLR9 was analyzed in two populations of immune system cells: monocytes and DCs (myeloid and plasmacytoid DCss) and a quantitative analysis of the presence of the tested receptor in the serum of patients diagnosed with IgAN and MPGN was undertaken. Then, the obtained results were evaluated in terms of correlation with morphological and biochemical parameters related to the functioning of the kidneys. Additionally, an analysis of the presence of EBV in the genetic material of GN patients was performed and its correlation with the level of TLR9 expression was recapped.

## 2. Results

### 2.1. Clinical Characteristic of the GN Patients and the Control Group

The research group and the control group consisted of 35 patients matched according to age (mean 37.1 ± 1.40) and gender (60% of recruited patients were men). In order to characterize the clinical features of both groups of patients, morphological assay was performed which showed no statistically significant abnormalities within WBC (white blood cells), LYM (lymphocytes), RBC (red blood cell), HGB (hemoglobin), PLT (platelets), serum creatinine, as well as serum immunoglobulin (Ig) M (IgM) and IgA and serum complement component C3 and C4 (Table 1). Significant changes were observed in the group of patients diagnosed with GN related to the presence of glomerular hematuria and arterial hypertension, which were 62.9% and 48.6%, respectively (Table 1). A total quantity of protein in a 24-h urine collection test was also recorded in the test group. Additionally, an increased level of urea (by 68.18%), BUN (blood urea nitrogen) (by 68.05%), and serum uric acid (by 20.95%) were determined in the studied patients as compared to the control group (Table 1). A significant decrease in the values of basic biochemical parameters in the study group was recorded as for eGFR (estimated glomerular filtration rate) (by 31.29%), serum IgG (by 50.32%), serum total protein (by 28.48%) and serum albumin (by 35.71%) (Table 1). 

#### Detailed Analysis of Morphological and Biochemical Parameters of Patients, Taking into Account the Disease Subunit: IgAN and MPGN

The next stage of our research was to characterize the group of patients based on the type of disease. It was shown that the research group consisted of 20 individuals diagnosed with IgAN and 15 patients diagnosed with MPGN (Table 2). From the obtained results, one can conclude that statistically significant differences within the analyzed morphological and biochemical parameters concerned an increase in LYM in patients with IgAN (by 52.12%) and a decrease in the total quantity of protein in a 24-h urine collection test (by 54.46%) relative to MPGN (Table 2). Additionally, a statistically significant difference was observed in the amount of EBV genetic material detected (*p* = 0.0026), which was higher in patients with MPGN than in IgAN (Table 2). Moreover, significant differences in frequencies of lymphocytes (*p* = 0.0003), amount of serum IgG (*p* = 0.019) and amount of serum complement C3 (*p* = 0.025) were noted in patients with these two entities.

Moreover, statistical analysis was performed concerning the diversity of the analyzed morphological and biochemical parameters of patients with IgAN, MPGN and those in the control group. The analysis pointed to statistically significant differences in parameters such as glomerular hematuria, arterial hypertension and total quantity of protein in a 24-h urine collection test. Moreover, changes in the values of LYM, RBC, HGB, eGFR, serum IgG, serum total protein, serum albumin were also observed, and they appeared to be lower than ones in the control group (Table 2).

### 2.2. Assessment of the Frequency of TLR9-Positive DCs and Monocytes

Another aspect of our research concerned establishing the frequency of TLR9-positive DCs and monocytes in both studied groups of patients. The conducted studies show that patients diagnosed with GN have a higher percentage of BDCA-1+CD19− and BDCA-2+CD123+ dendritic cells than patients in the control group (Table 3). In addition, the frequency of TLR9-positive both the BDCA-1+CD19− and BDCA-2+CD123+ DCs was higher in the studied patients as compared with the healthy persons, and was 3.85- and 2.89-fold higher, respectively. In case of monocytes, we observe a significant decrease in the incidence of CD14++CD16- classical monocytes and an increase in the fraction of intermediate monocytes. However, in both fractions tested, the incidence of TLR9-positive monocytes is higher in patients diagnosed with GN than in control group by 1.99 and 2.24 fold, respectively (Table 3). Moreover, statistically significant higher serum concentrations of TLR9 were observed in patients from the studied group as compared to the control group.

### 2.3. Detailed Analysis of the Frequencies of TLR9-Positive DCs and Monocytes of Patients TAking into Account the Disease Subunit (IgAN and MPGN)

**Figure 1:**
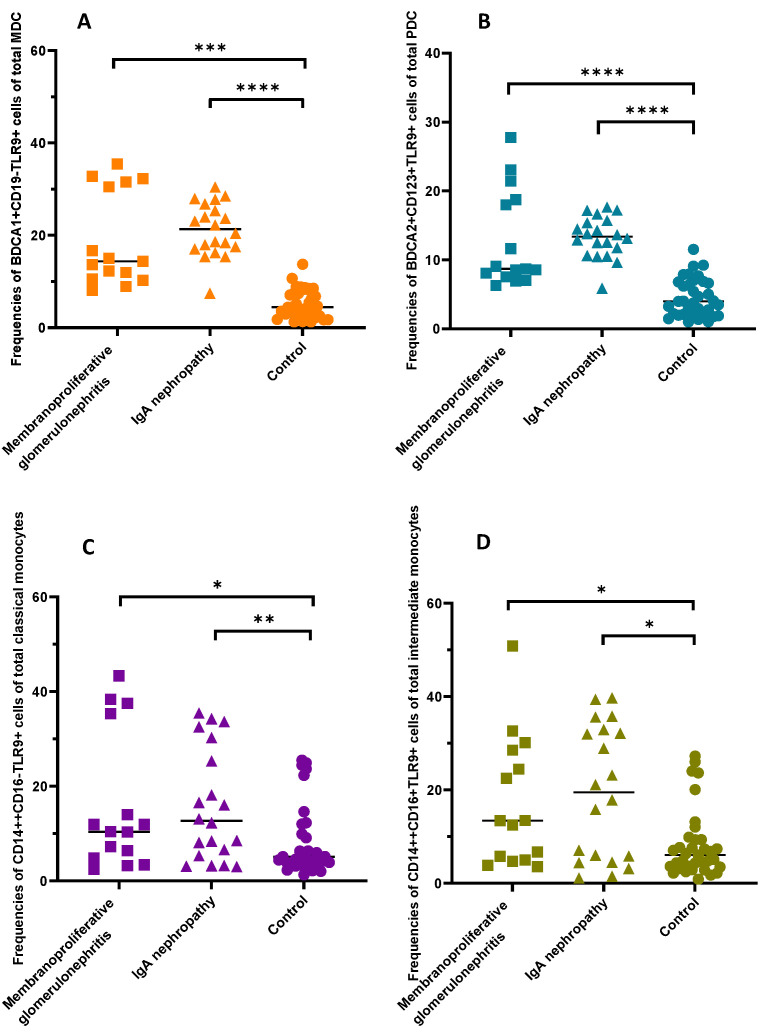
Frequencies of the DCs and monocytes expressing TLR9 antigen in the patients and controls. (**A**) Frequencies of BDCA1+CD19−TLR9+ cells of total mDC; (**B**) frequencies of BDCA2+CD123+TLR9+ cells of total pDC; (**C**) frequencies of CD14++CD16-TLR9+ cells of total classical monocytes; (**D**) frequencies of CD14++CD16+TLR9+ cells of total intermediate monocytes. (*, *p* < 0.05, **; *p* < 0.01, ***; *p* < 0.001; ****, *p* < 0.0001).

The next stage involved the original cytometric results analysis in terms of disease entities. However, no statistically significant differences were observed as for the tested parameters between patients with IgAN and those with MPGN (Table 4). Additionally, no statistically significant differences in serum TLR9 concentration were noted between the two analyzed groups of patients. Therefore, we have carried out the reference grouped by disease entities results of patients in relation to the control group. The analysis shows that the percentage of TLR9 on dendritic cells and monocytes is significantly higher in patients with IgAN and MPGN than in healthy subjects (Table 4) (Figure 1). Moreover, statistically significant serum concentrations of TLR9 were observed in patients with IgAN and MPGN in the control group (Table 4).

### 2.4. Assessment of EBV Prevalence in Patients with GN and the Controls

To further investigate the cause of the high percentage of TLR9 on monocytes and DCs, we searched for the presence of infectious agents including standard bacterial and fungal pathogens and several viruses. All tests performed were negative, except for the EBV virus. The PCR-based test pointed to the presence of EBV DNA copies/µg DNA in patients with a GN of 19.6 (0–54.4). Detailed analysis of the individual groups of patients included in the GN revealed the following values in patients with IgAN: 8.16 (0.00–24.3) copies/µg DNA and MPGN: 58.28 (0.00–221.7) copies/µg of DNA. Interestingly, the analysis of the serological status of the participants showed that all GN patients who were positive for EBV DNA had antibody titers indicative of viral reactivation assessed, as described by De Paschale et al. [17]. They were positive for anti-capsid antiviral (VCA) antigen IgG, IgM and IgA, anti-EBNA-1 IgG and IgA; and anti-early antigen (EA) IgA antibodies. On the other hand, control persons who were not found to have EBV DNA copies had anti-EBV status, suggesting past EBV infection [17]; positive only for anti-VCA IgG and anti-EBNA-1 IgG antibodies.

### 2.5. Analysis of the Correlation of TLR9 Concentration with Selected Biochemical Parameters, Immunophenotype and the Presence of EBV DNA

Spearman’s rank correlation analysis for TLR9 concentration was performed based on the obtained results of complete blood count, biochemical and immunological parameters. The results presented in Table 5 show that the concentration of TLR9 in the serum positively correlates with urea, serum uric acid, BUN (low correlation), total quantity of protein in a 24-h urine collection test (very high correlation) and frequencies of BDCA-1+CD19− and of BDCA-2+CD123+ DCs in the peripheral blood (moderate correlation). Moreover, a high positive correlation was also observed in the case of serum TLR9 concentration and the number of EBV copies. A negative correlation for eGFR (moderate correlation), serum IgG, serum total protein and serum albumin (high correlation) was observed. 

Additionally, the same analysis for patients diagnosed with GN was conducted. The results presented in Table 6 prove that serum TLR9 concentration positively correlates with the total quantity of protein in a 24-h urine collection test and the frequencies of BDCA-2+CD123+ DCs in the peripheral blood (moderate correlation), and the number of EBV DNA copies (very high correlation). On the other hand, a negative correlation was observed among this group of patients for the concentration of TLR9 in the serum and the serum IgG, and serum albumin (low correlation), and serum IgM (moderate correlation).

**Figure 2:**
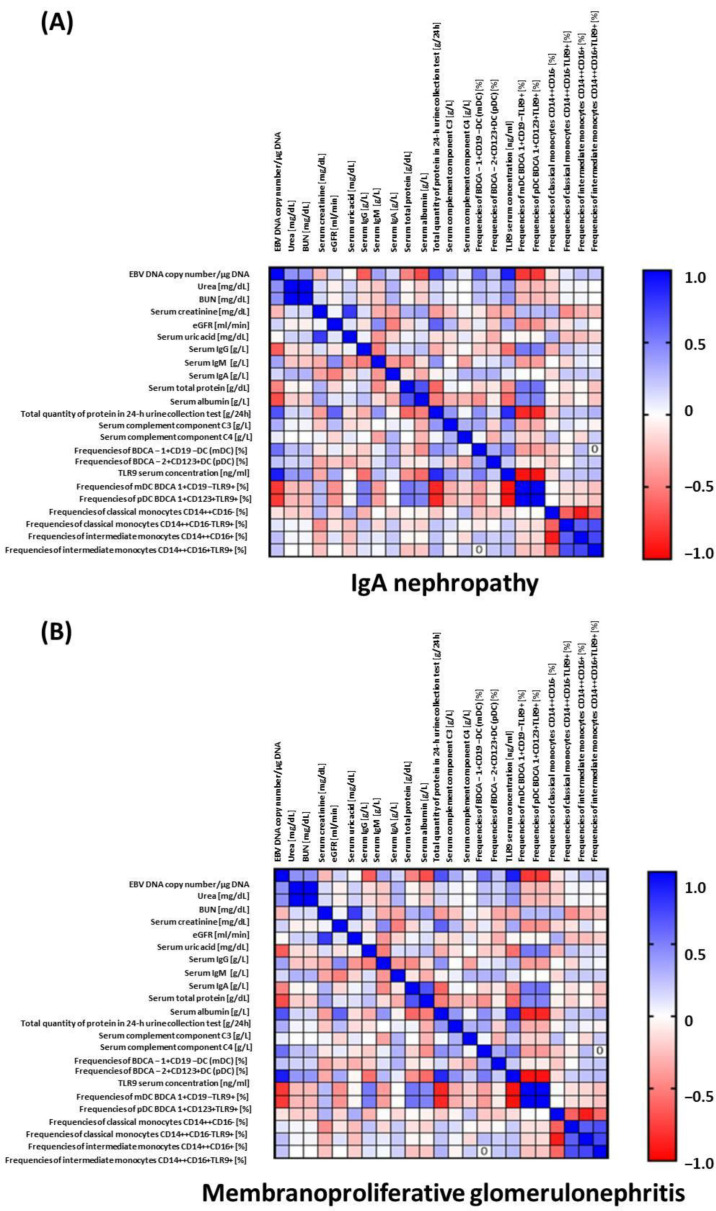
Correlation of the dendritic cells, monocytes and cell subsets expressing TLR9 antigen of IgAN (**A**) and MPGN (**B**) patients. Spearman’s rank coefficients were presented as intensity of the colors. The closer Rs is to +1 or −1, the stronger the correlation. A perfect positive correlation is +1 (blue color) and a perfect negative correlation is −1 (red color).

The detailed correlation of DCs, monocytes, and basic subsets of peripheral blood lymphocytes, and those subsets of cells expressing the TLR9 antigen of patients with IgAN (A) and MPGN (B) is presented in Figure 2. The analysis confirmed the associations of clinical parameters values in the disease process. In patients with IgAN, strong positive correlation between total quantity of protein in a 24-h urine collection test and TLR9 serum concentration (Spearman r = 0.84, *p* < 0.0001) but negative with frequencies of mDC BDCA1+CD19-TLR9+ in the peripheral blood (r = −0.85, *p* < 0.0001) and frequencies of pDC BDCA2+CD123+TLR9+ in the peripheral blood (r = −0.85, *p* < 0.0001) were all observed. Moreover, EBV DNA copy number/µg DNA was strongly, positively associated with TLR9 serum concentration (r = 0.89, *p* < 0.0001) but negatively with frequencies of mDC BDCA1+CD19-TLR9+ in the peripheral blood (r = −0.79, *p* < 0.0001) and frequencies of pDC BDCA2+CD123+TLR9+ in the peripheral blood (r = −0.78, *p* < 0.0001). In patients with MPGN, similar correlations were revealed regarding EBV DNA copy number/µg DNA which was strongly positively associated with TLR9 serum concentration (r = 0.98, *p* < 0.0001) but negatively with frequencies of mDC BDCA1+CD19-TLR9+ in the peripheral blood (r = −0.98, *p* < 0.0001) and frequencies of pDC BDCA2+CD123+TLR9+ in the peripheral blood (r = −0.98, *p* < 0.0001) and additionally negatively correlated with serum complement component C3 (r = −0.83, *p* < 0.0001). In patients with this entity, eGFR values and serum complement component C3 were positively correlated with frequencies of mDC BDCA1+CD19-TLR9+ in the peripheral blood (r = 0.73 and 0.77, respectively, *p* < 0.0001) and frequencies of pDC BDCA2+CD123+TLR9+ in the peripheral blood (r = 0.73 and 0.77, respectively, *p* < 0.0001) but negatively with TLR9 serum concentration (r = −0.73 and −0.77, respectively, *p* < 0.0001).

## 3. Discussion

As indicated in the literature and confirmed in our own observations, TLRs are a key element of non-specific responses that may be involved in the pathogenesis of kidney diseases [3,18,19]. They can influence the development and progression of kidney disease through many different mechanisms, including their activation by infectious agents, trauma or plant agonists (in the form of substances that bind to receptors and cause a cell reaction, usually in the form of immune complexes) that lead to overreaction or production of autoantibodies which may accumulate in the kidneys. Systemic activation of signaling pathways based on TLRs may also lead to the formation of numerous inflammatory mediators that may affect the progression of existing renal dysfunction [20,21]. Clinically, over-activation of TLRs can lead to ischemic kidney injury, acute kidney injury, end-stage renal failure, acute tubulointerstitial nephritis, acute kidney transplant rejection or delayed graft function, as well as the development and progression of glomerulopathy [3,18,19]. Our target TLR9s promote host defense by enhancing innate and adaptive immune responses that facilitate the migration of leukocytes (monocytes, lymphocytes) to areas of inflammation. Therefore, TLRs are often reported in the literature as a kind of threat sensors promoting the activation of not only cells of the immune system, but also internal kidney cells (podocytes). These cells are constantly exposed not only to many different compounds dissolved in the plasma, but also to the other dangers resulting from their unique location in the renal glomeruli [22,23]. Research to date on the role of TLR9in the pathogenesis of kidney disease is relatively scarce and focuses on acute kidney injury (AKI), chronic kidney disease (CKD) [24], systemic lupus erythematosus (SLE) [25], diabetic nephropathy [26] IgAN [27], and MPGN [28]. However, most of the published data concern the expression level of TLR9 on T and B lymphocytes and mainly involve studies in animal models (mouse models). In studies conducted by Summers et al. in 2010 it was shown that TLR9 is involved in the pathogenesis of SLE in a mouse model and could be a potential target in alleviating kidney damage in the early stages of the disease [29]. The studies conducted by Suzuki et al. in 2008 proved that TLR9 transcript levels correlated with MyD88 (Myeloid differentiation primary response 88) protein levels in mice with IgAN, which is involved in signaling in immune cells [30]. This protein acts as a kind of adapter, connecting the signals from the outside of the cell with proteins transmitting signals inside the cell via signaling pathways based on TLRs [31]. Additionally, they demonstrated that TLR9 transcription levels were associated with urinary albumin as well as serum IgA levels (*p* < 0.001 and *p* < 0.01, respectively) in mouse models [30]. Makita et al. in 2020 reported that TLR9s are also involved in the pathogenesis of IgAN in a mouse model. Researchers proved that activation of TLR9 following the injection of CpG-ODN into mice led to a deterioration in kidney function accompanied by the IgA and IgG deposits in the glomeruli. The researchers suggested that this is likely to be related to the increased levels of abnormally glycosylated IgA and IgG-IgA IC in the serum. In addition, they also showed that TLR9 activation was involved in Gd-IgA1 synthesis in human IgA1 secreting cell lines [27].

Similar studies were conducted in mouse models where the influence of TLRs on the development of MPGN was analyzed. Masum et al. in 2018 showed that the TLR9 receptor is overexpressed in murine podocytes (the BXSB/MpJ-Yaa mouse model) and causes damage to the renal glomeruli, which consequently led to the development of MPGN. Expression of TLR9 mRNA and its cytokines (IL1β, IL-6, IFN-γ and TNF-α) was significantly increased in glomeruli isolated from Yaa mice. In addition, the researchers showed that TLR9 expression was significantly correlated with anti-dsDNA antibody, proteinuria, indicators of renal function such as BUN and serum creatinine, as well as indicators of glomerular histopathology and parameters of podocyte damage (*p* < 0.05 and *p* < 0.01) [32]. 

The research carried out by our team shows that TLR9 receptors are involved in the development of IgAN and MPGN in humans, too. The analyses performed with the use of flow cytometry showed that the differences in the frequency of TLR9-positive DCs and monocytes in both studied groups of patients (IgAN and MPGN) were significant as compared to those in the control group. Patients diagnosed with GN had a higher percentage of BDCA-1+CD19− and BDCA-2+CD123+ DCs than patients in the control group. Moreover, the frequency of TLR9-positive both BDCA-1+CD19− and BDCA-2+CD123+ DCs was higher than in healthy persons (3.85 and 2.89 fold, respectively). In the case of monocytes, we observed a significant decrease in the incidence of CD14+CD16- classical monocytes and an increase in the fraction of intermediate monocytes. However, in both examined fractions the frequency of TLR9-positive cells is higher in patients diagnosed with GN than in control group (1.99 and 2.24 fold, respectively). Although no statistically significant differences in serum TLR9 levels were observed between the two analyzed groups of patients (IgAN and MPGN), there were significant differences between the two studied populations with the control group. Additionally, we showed that TLR9 serum concentration significantly correlates with EBV DNA copy number/µg DNA (*p* < 0.0001), serum IgG (*p* = 0.018), serum IgM (*p* = 0.013), serum albumin (*p* = 0.023), total quantity of protein in a 24-h urine collection test (*p* < 0.0001), and frequencies of BDCA-2+CD123+ DCs in the peripheral blood (pDC) (*p* = 0.014). The obtained results, especially in terms of the analysis of the correlation of TLR9 concentration with biochemical parameters, appear to be similar to the results obtained by researchers in mouse models. The reason for the targeting of EBV actions at the TLR9 may be its function of detecting unmethylated CpG motifs. This type of molecule is present early in the initial infection. It has also been suggested that the increase in TLR9 inhibits EBV reactivation and subsequent lytic replication by inducing chromatin remodeling, thereby disrupting the transcription of the immediate-early lytic gene EBV. The action of TLR9 for host cells is beneficial because of EBV replication, preventing lysis and death of the cell where the virus multiplies [33]. Avoiding an immune response by EBV is associated with its latent membrane protein 1 (LMP1), which can inhibit TLR9 expression. Scientific reports indicate that increased expression of LMP1 in B lymphocytes blocked the activity of the TLR9 promoter, mRNA and protein levels. In addition, studies suggest that TLR9 concentration is lower in EBV-positive patients than in EBV-negative patients, and the presence of early antigen (EA) in serum, correlating with decreased TLR9 expression, may indicate EBV reactivation in the study group [34]. 

We are fully aware of the fact that the presented research results cannot constitute unambiguous evidence confirming that TLR9 expressed on monocytes and DCs can constitute potential biomarker molecules of IgAN and MPGN development, due to the relatively small sample studied. However, we hope that the presented data will help other scientists to conduct future analyzes to decipher the role of TLRs (including TLR9) in regulating inflammation and impaired immune responses leading to kidney damage. We strongly believe that comprehensive research on the interaction of TLRs with environmental, genetic, commensal and pathogenic microorganisms as well as endogenous agonists will allow these receptors to be classified as new and effective biomarker molecules allowing early diagnosis of renal dysfunction, but also the development of new therapies of GN patients, and for the maintenance of normal immune homeostasis in the patient’s body.

## 4. Materials and Methods

### 4.1. Patients and Healthy Volunteers

The study material for this paper were peripheral blood (PB) samples from 35 randomly selected, newly diagnosed, previously untreated patients (21 male patients and 14 female patients) with primary GN and 35 healthy age- and sex-matched healthy volunteers (control group), 20 patients diagnosed with IgA nephropathy (13 male patients and 7 female patients) and 15 patients with MPGN (8 male patients and 7 female patients). This study included patients who did not use immunomodulators or hormonal preparations, did not receive blood transfusions, showed no signs of infection for at least 3 months, and also those who did not have autoimmune diseases or allergies. In addition, the criteria defined no history of oncological treatment, prior tuberculosis treatment, or other chronic conditions that might be associated with impaired humoral or cellular immunity. 

All patients had a complete blood count performed, along with kidney function assessment and the diagnosis for the presence of common bacterial, viral and fungal pathogens. No bacterial (aerobic and anaerobic) pathogens or fungi were identified in any of the samples using standard breeding methods.

Additionally, the absence of genetic material for hepatitis A virus (HAV), hepatitis B virus (HBV), hepatitis C virus (HCV), hepatitis D virus (HDV), hepatitis E virus (HEV), enteroviruses, adenoviruses, human immunodeficiency virus (HIV), herpes simplex virus 1 and 2 (HSV-1 and -2), cytomegalovirus (CMV), human papillomavirus (HPV), parvovirus B19, influenza virus, severe acute respiratory syndrome coronavirus 2 (SARS-CoV-2) or microorganisms such as *Borrelia burgdorferi*, *Chlamydia trachomatis*, *Chlamydia pneumoniae*, *Mycobacterium tuberculosis*, *Toxoplasma gondii*, *Ureaplasma* spp. or *Listeria* spp. was established.

The diagnosis of GN was performed based on the histological analysis of kidney biopsy specimens, including standard hematoxylin and eosin staining, and immunohistochemical staining of immune complexes according to classic criteria [35] and previously published methodology [36].

This study was approved by the Ethics Committee of Medical University of Lublin (Decision No. KE-0254/236/2020). A written informed consent regarding the use of blood for scientific purposes was obtained from all patients. This study was carried out according to the Declaration of Helsinki standards.

### 4.2. Isolation of PB Mononuclear Cells

PB mononuclear cells were isolated from patients with GN and the healthy controls. Venous blood samples (10 mL) were collected by venipuncture using sterile, EDTA tubes (S-Monovette, SARSTEDT, Aktiengesellschaft&Co., Numbrecht, Germany). Standard density gradient centrifugation was used (Gradisol L, Aqua Medica, Łódź, Poland) for the aseptic separation of PB mononuclear cells.

### 4.3. Flow Cytometry

**Figure 3:**
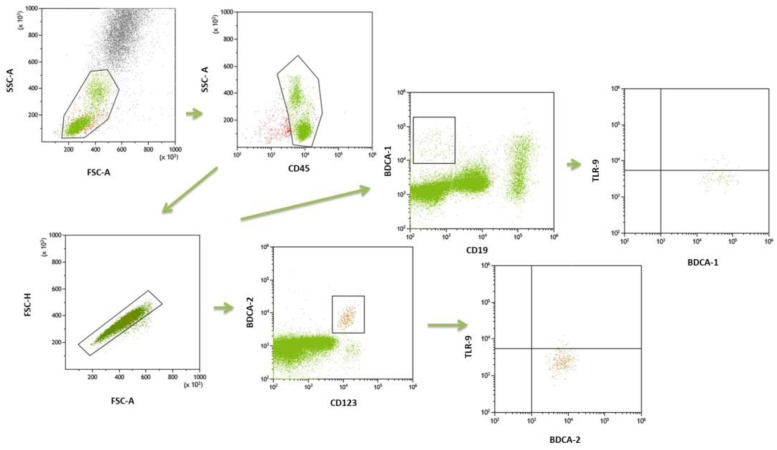
The gate strategy of dendritic cells.

**Figure 4:**
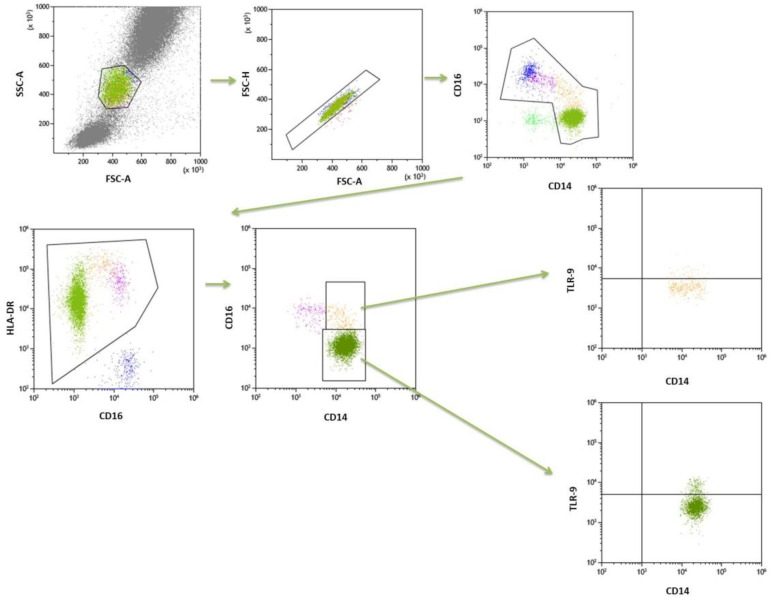
The gate strategy of monocytes.

Whole-blood samples were collected for immunophenotyping of mDC (BDCA1+CD19-), pDC (BDCA2+CD123+), classical monocytes (CD14++CD16-) and intermediate (CD14++CD16+) cells. The population of DCs was determined using 100 ul of blood samples and fluorescently labelled mouse monoclonal antibodies against the following cell surface markers: anti-BDCA-1 (CD1c) FITC (Biolegend, San Diego, CA, USA), anti-CD19 PeCy7 (Biolegend, San Diego, CA, USA), anti-CD45 V450 (BD Biosciences, San Jose, CA, USA), anti-BDCA-2 FITC (Biolegend, San Diego, CA, USA), anti-CD123 PeCy7 (Biolegend, San Diego, CA, USA). For immunophenotyping of monocytes, cells were stained with cell surface markers. The following fluorescently labeled mouse monoclonal antibodies were used: anti-CD14 FITC (BD Biosciences, San Jose, CA, USA), anti-CD16 V450 (BD Biosciences, San Jose, CA, USA), anti-HLA-DR Pe-Cy7 (BD Biosciences, San Jose, CA, USA). Monocytes were characterized by combining monoclonal antibodies and anti-HLA-DR as an additional gating step. Blood samples were incubated for 20 min in the dark with monoclonal antibodies. Afterwards, all samples were treated with lysis buffer (Lysing Buffer, BD Pharm Lyse, San Jose, CA, USA) for 15 min and washed in PBS solution (Sigma-Aldrich, Saint Louis, MO, USA). Cells were then incubated with anti-TLR9 APC (BD Biosciences, San Jose, CA, USA), permeabilized in FACS permeabilizing solution (BD Biosciences, San Jose, CA, USA) for 20 min and washed in PBS solution. The samples were examined in a Cytoflex LX (Beckman Coulter, Brea, CA, USA) and analyzed using the Kaluza Analysis program. The gate strategy is shown in Figure 3 and Figure 4.

### 4.4. Determination of TLR9 Concentration in the Patients Serum by ELISA Test

The commercial enzyme-linked immunosorbent assay (ELISA) kit Human Toll Like Receptor 9 (TLR9) (MyBioSource), with a sensitivity up to 0.06 ng/mL and assay range 20 ng/mL–0.312 ng/mL, was used for the quantitative determination of human TLR9 in plasma samples. For plasma separation, 5 mL samples of PB collected into EDTA tubes were used. Plasma samples were stored in liquid nitrogen until the time of analysis. The analysis was performed in accordance with the manufacturer’s recommendations. The ELISA Reader VictorTM3 (PerkinElmer, Waltham, MA, USA) microplate reader was used for measurements.

### 4.5. DNA Isolation and Calculation of EBV Load and Assessment of Anti-EBV Antibody Status

DNA was isolated from five million PBMCs using the QIAamp DNA Blood Mini Kit (QIAGEN, Hilden, Germany) according to the manufacturer’s instructions, and the number of EBV-specific DNA copies was calculated using the ISEX variant of the EBV PCR kit (GeneProof, Brno, Czech Republic). The concentration and purity of the isolated DNA was verified with a BioSpec-nano spectrophotometer (Shimadzu, Kyoto, Japan). All samples were analyzed in duplicate and a negative control (DNA elution buffer) was included. A specific conserved DNA sequence for the EBV nuclear antigen 1 gene (EBNA-1) was amplified using a 7300 Real-Time PCR System (Applied Biosystems, Foster City, CA, USA). Viral DNA copy number/μL eluent was adjusted to the DNA isolation efficiency and was expressed as viral DNA copy number/μg DNA. Due to the detection limit of ten EBV DNA copies/μL, all samples below this threshold were considered EBV negative (control group). The serological status of anti-EBV antibodies was assessed using ELISA kits (IBL International, Hamburg, Germany) on Victor TM3 (PerkinElmer, Waltham, MA, USA). IgA, IgM and IgG antibodies recognizing early antigen (EA), viral capsid antigen (VCA) and EBNA-1 were measured and the manufacturer specified cut-off values were applied [37].

### 4.6. Statistical Analysis

The statistical characteristics of continuous variables is presented as medians, inter-quartile ranges (IQRs) and arithmetic means with their standard deviations (SD). Discrete variables are presented as numbers and a percentage. The Shapiro–Wilk test was used to test the normality of continuous variables. Student’s *t*-test was used for comparisons between independent variables, and the Mann–Whitney U test was used for intergroup comparisons. The power and direction of relationships between pairs of continuous variables were determined using Spearman’s coefficient of rank correlation. The distributions of discrete variables in groups were compared with the Pearson’s chi-square test or Fisher’s exact test. The error was set at 5% and significance at *p*-value < 0.05. The statistical analysis was carried out with TibcoStatistica 13.3 (StatSoft, Palo Alto, CA, USA). The graphs were generated with the use of GraphPad Prism (GraphPad Prism Software v. 9.4.1 (687), San Diego, CA, USA).

## 5. Conclusions

In our study, we presented the role of TLR9 in the immunopathogenesis of the two most common types of GN, i.e., IgAN and MPGN. We pointed to statistically significant differences between the studied disease subunits in the context of selected morphological and biochemical parameters, as well as their differentiation in terms of the expression of TLR9on monocytes and DCs as compared to control group. Although no statistically significant differences in serum TLR9 levels were observed between the two analyzed groups of patients (IgAN and MPGN), there were significant differences between the two studied populations with the control group. Additionally, we showed that TLR9 serum concentration significantly correlates with EBV DNA copy number/µg DNA, serum IgG, IgM, serum albumin, total quantity of protein in a 24-h urine collection test and frequencies of BDCA-2+CD123+ DCs (pDC) in the peripheral blood. The presented results of our research may suggest that one of the factors influencing the pathogenesis of IgAN and MPGN may be EBV reactivation and its influence on the involvement of the innate response through the use of TLRs (in our case by TLR9). We also hope that the presented data will not only enrich literature reports on the pathogenesis of GN, but also help other scientists to conduct research aimed at clarifying the role of TLRs (including TLR9) in the regulation of inflammation and an impaired immune response resulting in kidney damage in humans.

## Figures and Tables

**Table 1:** Clinical characteristics of the GN patients and the control group.

Parameter	GN Patients (*n* = 35)		Healthy Control Group (*n* = 35)		*p* Value
	Mean ± SD	Median (IQR)	Mean ± SD	Median (IQR)	
Age [years]	37.1 ± 1.40	35.0 (26.0–45.0)	37.1 ± 1.40	35.0 (26.0–45.0)	1.0
Male [%]	21 (60)		21 (60)		1.0
Glomerular hematuria (%)	22 (62.9)		0 (0)		<0.0001 *
Arterial hypertension (%)	17 (48.6)		0 (0)		<0.0001 *
WBC [10^3^/mm^3^]	7.0 ± 1.8	6.7 (5.3–8.6)	6.71 ± 0.6	6.77 (6.4–7.2)	0.96
LYM [10^3^/mm^3^]	2.1 ± 0.8	1.98 (1.5–2.6)	2.48 ± 0.8	2.44 (1.9–2.9)	0.069
RBC [10^6^/mm^3^]	4.5 ± 1.3	4.36 (4.1–4.7)	4.88 ± 0.6	4.79 (4.4–5.4)	0.00081 *
HGB [g/dL]	13.1 ± 1.5	13.2 (12.2–14.0)	14.17 ± 1.0	14.14 (13.4–15.1)	0.00075 *
PLT [10^3^/mm^3^]	240.2 ± 57.4	223.0 (197.0–277.0)	263.14 ± 55.7	263.0 (216.0–312.0)	0.07
Urea [mg/dL]	51.8 ± 30.6	40.9 (26.6–78.0)	30.8 ± 6.0	32.0 (28.0–35.8)	0.0062 *
BUN [mg/dL]	24.2 ± 14.3	19.1 (12.4–36.4)	14.4 ± 2.8	14.95 (13.1–16.7)	0.0062 *
Serum creatinine [mg/dL]	1.2 ± 0.6	0.96 (0.8–1.8)	0.95 ± 0.1	0.97 (0.85–1.1)	0.28
eGFR [mL/min]	86.2 ± 35.2	81.5 (56.5–117.5)	125.46 ± 9.7	122.36 (118.0–133.4)	<0.0001 *
Serum uric acid [mg/dL]	7.1 ± 1.8	7.5 (5.4–8.2)	5.87 ± 1.3	5.8 (4.6–7.3)	0.0016 *
Serum IgG [g/L]	6.2 ± 3.0	5.7 (4.2–6.9)	12.48 ± 1.9	12.7 (11.4–13.9)	<0.0001 *
Serum IgM [g/L]	1.7 ± 1.1	1.3 (0.8–2.9)	1.58 ± 0.4	1.57 (1.3–1.8)	0.63
Serum IgA [g/L]	2.4 ± 1.7	1.9 (1.0–3.4)	2.39 ± 0.8	2.51 (1.8–3.0)	0.29
Serum total protein [g/dL]	5.3 ± 0.9	5.3 (4.8–6.1)	7.41 ± 0.5	7.6 (6.95–7.8)	<0.0001 *
Serum albumin [g/L]	2.7 ± 0.9	2.8 (2.1–3.4)	4.20 ± 0.4	4.29 (3.9–4.5)	<0.0001 *
Total quantity of protein in a 24-h urine collection test [g/24 h]	3.8 ± 3.4	3.7 (0.3–7.0)	N/A	N/A	N/A
Serum complement component C3 [g/L]	1.2 ± 0.4	1.2 (1.0–1.3)	1.28 ± 0.2	1.25 (1.2–1.4)	0.32
Serum complement component C4 [g/L]	0.30 ± 0.08	0.27 (0.25–0.35)	0.27 ± 0.1	0.28 (0.2–0.3)	0.42

Abbreviations: GN (primary glomerulonephritis), WBC (white blood cells), LYM (lymphocytes), RBC (red blood cell), HGB (hemoglobin), PLT (platelets), BUN (blood urea nitrogen), eGFR (estimated glomerular filtration rate), and N/A (not applicable). * Statistically significant, *p* value < 0.05.

**Table 2:** Differentiation of morphological and biochemical parameters among patients with GN and the control group.

Parameter	IgAN (*n* = 20)	MPGN (*n* = 15)	Healthy Control group (*n* = 35)	*p* Value
	Median (IQR)	Median (IQR)	Median (IQR)	
Age [years]	42.5 (31.5–55.5)	27.0 (25.0–36.0)	35.0 (26.0–45.0)	0.030 *
Male [%]	13 (65.0)	8 (53.3)	21 (60)	0.78
Glomerular hematuria (%)	11 (55.0)	11 (73.3)	0 (0)	<0.0001 *
Arterial hypertension (%)	8 (40.0)	9 (60.0)	0 (0)	<0.0001 *
WBC [10^3^/mm^3^]	6.75 (5.7–9.0)	6.57 (5.1–8.4)	6.77 (64–72)	0.66
LYM [10^3^/mm^3^]	2.43 (1.9–3.0)	1.57 (1.3–1.98)	2.44 (19–29)	0.0005 *
RBC [10^6^/mm^3^]	4.35 (4.0–4.6)	4.4 (4.1–4.9)	4.79 (44–54)	0.0027 *
HGB [g/dL]	13.3 (12.5–14.2)	13.0 (12.1–13.5)	14.14 (134–151)	0.0019 *
PLT [10^3^/mm^3^]	243.0 (209.5–272.5)	212.0 (177.0–304.0)	263.0 (2160–3120)	0.11
Urea [mg/dL]	38.93 (26.8–75.5)	49.5 (26.0–78.3)	32.0 (280–358)	0.023 *
BUN [mg/dL]	18.19 (12.5–35.3)	23.13 (12.1–36.6)	14.95 (131–167)	0.023 *
Serum creatinine [mg/dL]	1.04 (0.8–2.2)	0.96 (0.7–1.8)	0.97 (085–11)	0.50
eGRF [ml/min]	85.11 (66.2–120.4)	81.54 (53.3–116.3)	122.36 (1180–1334)	<0.0001 *
Serum uric acid [mg/dL]	7.6 (5.8–8.3)	7.1 (5.0–8.2)	5.8 (46–73)	0.0057
Serum IgG [g/L]	6.35 (5.2–8.5)	4.24 (3.0–6.1)	12.7 (114–139)	<0.0001 *
Serum IgM [g/L]	1.37 (1.0–2.8)	1.2 (0.5–3.1)	1.57 (13–18)	0.73
Serum IgA [g/L]	1.96 (1.4–4.1)	1.39 (0.6–3.1)	2.51 (18–30)	0.26
Serum total protein [g/dL]	5.3 (4.9–6.2)	5.4 (4.4–6.0)	7.6 (695–78)	<0.0001 *
Serum albumin [g/L]	2.9 (2.3–3.5)	2.3 (1.9–3.1)	4.29 (39–45)	<0.0001 *
Total quantity of protein in a 24-h urine collection test [g/24 h]	0.78 (0.1–4.4)	5.7 (3.0–8.0)	N/A	<0.0001 *
Serum complement component C3 [g/L]	1.24 (1.1–1.4)	1.18 (0.7–1.3)	1.25 (12–14)	0.14
Serum complement component C4 [g/L]	0.27 (0.2–0.4)	0.27 (0.25–0.3)	0.28 (02–03)	0.63

Abbreviations: GN (primary glomerulonephritis), IgAN (IgA nephropathy), MNGN (membranoproliferative glomerulonephritis), WBC (white blood cells), LYM (lymphocytes), RBC (red blood cell), HGB (hemoglobin), PLT (platelets), BUN (blood urea nitrogen), eGFR (estimated glomerular filtration rate), Ig (immunoglobulin), N/A (not applicable). * Statistically significant, *p* value < 0.05.

**Table 3:** Frequencies of TLR9-positive DCs and monocytes among patients from the research group and the control group.

Parameter	GN Patients (*n* = 35)		Healthy Control Group (*n* = 35)		*p* Value
	Mean ± SD	Median (IQR)	Mean ± SD	Median (IQR)	
Frequencies of BDCA-1+CD19− DCs in the peripheral blood (mDC) [%]	1.05 ± 0.4	1.0 (0.8–1.2)	0.55 ± 0.3	0.50 (0.3–0.7)	<0.0001 *
Frequencies of BDCA-2+CD123+ DCs in the peripheral blood (pDC) [%]	0.49 ± 0.4	0.42 (0.2–0.7)	0.23 ± 0.2	0.17 (0.1–0.3)	0.00022 *
mDC/pDC ratio	4.87 ± 0.89	2.1 (1.8–5.1)	8.73 ± 1.71	2.53 (1.5–6.6)	0.61
Frequencies of mDC BDCA1+CD19-TLR9+ of total mDC [%]	20.24 ± 7.9	18.4 (14.4–27.8)	5.26 ± 3.1	4.47 (2.6–8.2)	<0.0001 *
Frequencies of pDC BDCA2+CD123+TLR9+ of total pDC [%]	13.07 ± 5.0	12.6 (8.7–16.7)	4.52 ± 2.7	3.99 (2.0–6.8)	<0.0001 *
Frequencies of classical monocytes CD14++CD16- in the peripheral blood [%]	85.82 ± 4.7	87.07 (80.9–89.1)	90.63 ± 4.2	91.8 (88.3–93.9)	<0.0001 *
Frequencies of classical monocytes CD14++CD16-TLR9+ of total classical monocytes [%]	15.92 ± 12.9	11.89 (5.3–30.3)	7.99 ± 7.3	5.07 (3.4–9.9)	0.0036 *
Frequencies of intermediate monocytes CD14++CD16+ in the peripheral blood [%]	9.96 ± 4.9	8.49 (6.0–13.9)	6.23 ± 3.1	5.68 (3.5–8.5)	0.00089 *
Frequencies of intermediate monocytes CD14++CD16+TLR9+ of total [%]	18.46 ± 1.39	15.83 (5.0–32.0)	8.24 ± 7.3	6.04 (3.5–9.4)	0.0024 *
TLR9 serum concentration [ng/mL]	32.14 ± 1.31	30.4 (18.6–45.9)	4.78 ± 2.8	4.29 (2.9–7.2)	<0.0001 *

Abbreviations: CD (cluster of differentiation), DCs (dendritic cells), mDC (myeloid dendritic cells), pDC (plasmacytoid dendritic cells), TLR9 (Toll-like receptor 9). * Statistically significant, *p* value < 0.05.

**Table 4:** Differentiation of TLR9 expression on dendritic cells and monocytes among patients in the research group, taking into account the IgAN and MPGN, and the control group.

Parameter	IgAN(*n* = 20)	MPGN (*n* = 15)	Healthy Control Group (*n* = 35)	*p* Value
	Median (IQR)	Median (IQR)	Median (IQR)	
Frequencies of BDCA-1+CD19− DC in the peripheral blood (mDC) [%]	1.05 (0.8–1.2)	0.9 (0.8–1.3)	0.50 (03–07)	<0.0001 *
Frequencies of BDCA-2+CD123+ DC in the peripheral blood (pDC) [%]	0.42 (0.2–0.6)	0.4 (0.3–0.9)	0.17 (01–03)	0.0009 *
mDC/pDC ratio	2.14 (1.6–5.2)	2.1 (1.8–4.7)	2.53 (15–66)	0.82
Frequencies of mDC BDCA1+CD19-TLR9+ of total mDC [%]	21.34 (17.3–26.1)	14.35 (10.5–31.5)	4.47 (26–82)	<0.0001 *
Frequencies of pDC BDCA2+CD123+TLR9+ of total pDC [%]	13.34 (11.2–15.6)	8.7 (7.6–18.8)	3.99 (20–68)	<0.0001 *
Frequencies of classical monocytes CD14++CD16- in the peripheral blood [%]	86.98 (81.4–89.3)	87.1 (80.9–89.1)	91.8 (883–939)	0.0002 *
Frequencies of classical monocytes CD14++CD16-TLR9+ of total classical monocytes [%]	12.68 (5.9–27.8)	10.4 (4.9–35.4)	5.07 (34–99)	0.014 *
Frequencies of intermediate monocytes CD14++CD16+ in the peripheral blood [%]	8.53 (6.4–12.6)	7.7 (6.0–16.6)	5.68 (35–85)	0.0038 *
Frequencies of intermediate monocytes CD14++CD16+TLR9+ of total intermediate [%]	19.49 (5.2–32.6)	13.4 (5.0–28.5)	6.04 (35–94)	0.0098
TLR9 serum concentration [ng/mL]	28.21 (22.1–34.3)	45.9 (16.2–49.8)	4.29 (29–72)	<0.0001 *

Abbreviations: CD (cluster of differentiation), DCs (dendritic cells), mDC (myeloid dendritic cells), pDC (plasmacytoid dendritic cells), and TLR9 (Toll-like receptor 9). * Statistically significant, *p* value < 0.05.

**Table 5:** Correlation between serum TLR9 concentration and selected biochemical and immunological parameters of the studied population.

Parameter	TLR9 Serum Concentration [ng/mL] (*n* = 70)		
	Spearman R	t(N−2)	*p* Value
EBV DNA copy number/µg DNA	0.79	10.65	<0.0001 *
Urea [mg/dL]	0.30	2.62	0.011 *
BUN [mg/dL]	0.30	2.62	0.011 *
Serum creatinine [mg/dL]	0.07	0.60	0.55
eGFR [ml/min]	−0.52	−5.05	<0.0001 *
Serum uric acid [mg/dL]	0.35	3.06	0.0031 *
Serum IgG [g/L]	−0.73	−8.83	<0.0001 *
Serum IgM [g/L]	−0.23	−1.94	0.057
Serum IgA [g/L]	−0.12	−1.00	0.32
Serum total protein [g/dL]	−0.76	−9.70	<0.0001 *
Serum albumin [g/L]	−0.74	−9.13	<0.0001 *
Frequencies of BDCA-1+CD19− DC in the peripheral blood (mDC) [%]	0.55	5.50	<0.0001 *
Frequencies of BDCA-2+CD123+ DC in the peripheral blood (pDC) [%]	0.41	3.75	0.0004 *

Abbreviations: EBV (Epstein–Barr virus), BUN (blood urea nitrogen), eGFR (estimated glomerular filtration rate), Ig (immunoglobulin), and N/A (not applicable). * Statistically significant, *p* value < 0.05; Spearman’s rank order correlations.

**Table 6:** Correlation between serum TLR9 concentration and selected morphological, biochemical and immunological parameters for patients diagnosed with GN.

Parameter	TLR9 Serum Concentration [ng/mL] (*n* = 35)		
	Spearman R	t(N−2)	*p* Value
EBV DNA copy number/µg DNA	0.92	13.06	<0.0001 *
Urea [mg/dL]	0.10	0.59	0.56
BUN [mg/dL]	0.10	0.59	0.56
Serum creatinine [mg/dL]	−0.17	−1.01	0.32
eGFR [ml/min]	−0.28	−1.65	0.11
Serum uric acid [mg/dL]	0.19	1.10	0.28
Serum IgG [g/L]	−0.40	−2.49	0.018 *
Serum IgM [g/L]	−0.42	−2.63	0.013 *
Serum IgA [g/L]	−0.22	−1.27	0.21
Serum total protein [g/dL]	−0.19	−1.12	0.27
Serum albumin [g/L]	−0.38	−2.38	0.023 *
Total quantity of protein in a 24-h urine collection test [g/24 h]	0.61	4.46	<0.0001 *
Frequencies of BDCA-1+CD19− DC in the peripheral blood (mDC) [%]	0.18	1.03	0.31
Frequencies of BDCA-2+CD123+ DC in the peripheral blood (pDC) [%]	0.41	2.59	0.014 *

Abbreviations: EBV (Epstein–Barr virus), BUN (blood urea nitrogen), eGFR (estimated glomerular filtration rate), Ig (immunoglobulin), and N/A (not applicable). * Statistically significant, *p* value < 0.05; Spearman’s rank order correlations.

## Data Availability

The data presented in this study are available on request from the authors.
